# Production of novel theranostic nano-vector based on superparamagnetic iron oxide nanoparticles/miR-497 targeting colorectal cancer

**DOI:** 10.1038/s41598-025-88165-3

**Published:** 2025-02-04

**Authors:** Asmaa M. Elfiky, May M. Eid, May El-Manawaty, Zeinab A. Elshahid, Elham Mohamed Youssef, Khaled Mahmoud

**Affiliations:** 1 Environmental and Occupational Medicine Department, Environment and Climate Change Research Institute, National Research Centre, Cairo, Egypt; 2 Physics Institute, National Research Center, Dokki, Cairo, Egypt; 3 Pharmaceutical Sciences Institute, Department of Pharmacognosy, National Research Centre, Cairo, Egypt; 4 Chemistry of Natural and Microbial Products, Pharmaceutical Industry Research Institute, National Research Centre, Cairo, Egypt; 5 Biochemistry Department, National Research Centre, Cairo, Egypt

**Keywords:** Colorectal cancer, SPION@Ag@Cs nano-carrier, miR-497-5p, PD1/PDL1, CTLA-4 oncogenes, Biophysics, Biotechnology, Cancer, Computational biology and bioinformatics, Drug discovery, Immunology, Molecular biology

## Abstract

Colorectal cancer (CRC) is a serious public health concern worldwide. Immune checkpoint inhibition medication is likely to remain a crucial part of CRC clinical management. This study aims to create new super paramagnetic iron oxide nano-carrier (SPION) that can effectively transport miRNA to specific CRC cell lines. In addition, evaluate the efficiency of this nano-formulation as a therapeutic candidate for CRC. Bioinformatics tools were used to select a promising tumor suppressor miRNA (mir-497-5p). Green route, using *Fusarium oxyporium* fungal species, manipulated for the synthesis of SPION@Ag@Cs nanocomposite as a carrier of miR-497-5p. That specifically targets the suppression of PD1/PDL1 and CTLA4pathways for colorectal therapy. UV/visible and FTIR spectroscopy, Zeta potential and MTT were used to confirm the allocation of the miR-497 on SPION@Ag@Cs and its cytotoxicity against CRC cell lines. Immunofluorescence was employed to confirm transfection of cells with miR-497@NPs, and the down- regulation of CTLA4 in HT29, and Caco2 cell lines. On the other hand, PDL1 showed a significant increase in colorectal cell lines (HT-29 and Caco-2) in response to mir497-5p@Nano treatment. The data suggest that the mir-497 -loaded SPION@Ag@Cs nano-formulation could be a good candidate for the suppression of CTLA4in CRC human cell lines. Consequently, the targeting miR-497/CTLA4 axis is a potential immunotherapy treatment strategy for CRC.

## Introduction

Of the top 10 diseases diagnosed worldwide, colorectal cancer (CRC) is the third most frequent; 1.25 million people receive a colorectal cancer diagnosis annually^1^, and the illness claims the lives of more than 600,000 people each year^2,3^. Chemotherapy, radiation therapy, and surgical resection are the usual treatments for colorectal cancer. However according to reports, over half of patients who receive severe chemotherapy and surgical resection experience a disease recurrence^4^ and after five years, the survival rate is still quite low, at roughly 60%. Consequently, to improve patient prognosis, additional options are required despite the availability of all currently recommended medicines^5^.

MicroRNAs, an endogenous type of non-coding RNAs, are essential for numerous cellular functions, such as differentiation and proliferation. References^6–9^. Notably, studies on the dysregulation of miRNA in malignancies, including colon cancer^10–12^. miR-497, a cancer metastasis-related miRNA, is dysregulated in various malignancies, including CRC^13^. Also, miR-497 inhibits different genes related to CRC cell invasion, such as insulin-like growth factor 1 receptor, Fos-related antigen-1 (Fra-1)^14^. Zhang et al.^15^ reported that the expression of miR-497 is downregulated in different types CRC cell lines including (LoVo, RKO, HCT15, HCT28, HCT116, and SW480), compared with the normal fetal human colon epithelial cell line (CRL-1831). As a result, restoration of miR-497 expression can reduce tumor growth and proliferation.

The three oncogenes, Cytotoxic T Lymphocyte Associated Antigen 4 (CTLA-4), Programmed death-ligand 1 (PD-L1), and Programmed Cell Death Protein 1 (PD1), were primarily concerned with promoting the growth of colorectal cancer (CRC), worsening inflammatory reactions, and metastasis through the secretion or activation of cytokines and chemokines in the tumor microenvironment^16–19^.

Previously, we created a super-paramagnetic monodisperse γFe2O3@Ag nanocomposite utilizing a green method with the fungus Fusarium Oxyporium. Because of the natural synthesis process, the size has been determined as less than 10 nm, and the NPs are naturally covered in a chitosan outermost shell. It was discovered that these nanocomposites were fluorescent at various excitation wavelengths. In vitro*,* cytotoxicity tests revealed that at 100 μg/ml, γFe2O3@Ag@Cs caused less than 10% of the cancer cell lines in the prostate (PC3), liver (HepG2), colon (HT116), and lung (A549). Furthermore, in vivo experiments on mice have shown that NPs up to 100 mg/kg had no discernible effects on the counts of immune cell subsets or the functions of the liver and kidneys. Furthermore, the liver, kidney, brain, spleen, heart, and lung were found to have normal architecture upon histopathological examination (data in publication). FTIR imaging was also performed on paraffin-embedded tissues from mice administered 25, 50, and 100 mg/kg γFe2O3@Ag NPs^20^.

Studies conducted in vitro*, *in vivo*,* and ex vivo have suggested the use of γFe2O3@Ag@Cs NPs as a safe immunomodulator to boost the benefits of vaccine adjuvants. We confirmed this theory^21^ using the complementation of in vivo, in vitro, and ex vivo studies on BALB/c male mice obtained from the Laboratory Animal Center at NRC to determine the cell toxicity, uptake, and immunological response of the proposed core–shell NPs by various organs and cells. Our results demonstrated an increase in the level of nucleic acids and signaling transduction synchronized with an increase in the release of IgG and IgM cytokines. These data could be evidence that γFe2O3@Ag NPs could be employed as immuno-modulator^22,23^.

This study proposes a theranostic strategy based on targeted γFe2O3@Ag@Cs NPs. A vector encoding for tumor suppressor miRNA has been incorporated into the nanocomposite. This vector maintains stable over-expression of the mature and functional miRNA inside the transfected cells and tissues. Using integrated in vitro experiments, the binding and transfection efficiency of the Nanocomposite + anti-CRC miRNA nano-complex was assessed. In addition, the therapeutic efficacy of this nano-formulation was assessed.

## Material and methods

### Preparation of microRNA expression vector

Using the QIAamp genomic DNA kit (QIAGEN, USA) for isolating human PBMC genomic DNA. The pri-miR-497-5p sequence was amplified by Hotstartaq PCR master mix (QIAGEN, USA) from extracted genomic DNA. The primers for the PCR amplification were tcga-ggatcc-aagtttgtcacaaggcccca (forward) and tcga-gctagc-ggtcttcccagcactgctat (reverse).

### Recombinant vector constructs and sequence verification

The PCR was performed as follows: 5 min at 95 °C and 35 cycles of 15 s at 95 °C, 30 s at 56 °C plus 30 s at 72 °C. Then, the purified PCR product and pCMV-miR vector Origene (Rockville, MD, USA) were digested and PCR product ligated into pCMV-miR to generate pmiR-497 using T4 DNA ligase (Thermo Scientific, USA). Subsequently, pmiR-497 construct was transformed into Escherichia coli TOP10 cells and then, purified by an endotoxin-free GeneJET Plasmid Maxprep Kit (Thermo Scientific, USA), according to the manufacturer’s protocol. The pNull was used here as a control vector. All constructs were verified by sequencing.

### Spectroscopic analysis

#### UV–Visible analysis

The evaluation of loading efficiency and the amount of miRNA loaded in nano-plexes were examined using UV–Visible spectroscopy to examine the formation of bonding to select the best condition of preparation. Optical properties were analyzed using ultraviolet–visible (UV–Vis) absorption spectrophotometer (JASCO V-630) in the range 200–800 nm with 0.2 nm resolution.

#### Fourier transform infrared (FTIR) spectroscopy analysis

Fourier transform infrared (FTIR) spectroscopy analysis is performed to study the functional groups on the most outer layer and responsible for NPs bonding. Attenuated Total Refectance Fourier transform infrared (ATR-FTIR) spectroscopy was used to investigate the structure of the prepared samples using diamond cell using a Bruker Vertex 80 with 4 cm − 1 resolution.

#### Particle size analysis

The particle size distribution and zeta potential has been determined by the dynamic light scattering (DLS) technique. The average particle size and size distribution were assessed using the dynamic light scattering (DLS) approach. The following were the measuring parameters: The refractive index is 1.333, and the viscosity is 0.933 cp.

### Cytotoxic activity

#### Cell culture

The human colorectal (HT-29 and Caco-2) cancer cell lines were maintained in DMEM-F12 medium. The media was supplemented with 10% foetal bovine serum at 37 °C in 5% CO_2_ and 95% humidity. Cells were sub-cultured using trypsin versene 0.15%. All cell lines were kindly provided by Professor Stig Linder, Oncology and Pathology department, Karolinska Institute, Stockholm, Sweden, originally obtained from ATCC.

#### Cytotoxicity on cancer monolayers

The cytotoxic assay on cancer cell line done according to the method of with slight modifications. Briefly, after 24 h of seeding 20,000 cells per well for HCT-116, HT-29 cell lines (in 96 well plates), the medium was changed to fresh medium and cells were treated with 100 μg/ml final concentration of the nanocomposite in triplicates for 48 h. 100 μM doxorubicin was used as positive control and 0.5% DMSO was used as negative control. Cell viability was determined using the MTT (3- (4, 5-dimethylthiazol-2-yl)-2,5-diphenyltetrazolium bromide) assay as described previously.

The percent cytotoxicity was calculated according to the following equation:

$$\% {\text{cytotoxicity}} = \left[ {{1} - \, \left( {{\text{AVx}}/{\text{AVNC}}} \right)} \right] \times {1}00$$

where AV: average, X: absorbance of sample well, NC: absorbance of negative control measured at 595 nm with reference at 690 nm.

### Immunohistochemistry

Cells were washed with PBS and fixed for 10 min at room temperature with 4% paraformaldehyde, permeabilized for 10 min at room temperature with 0.25% triton-X 100 in PBS, washed twice with PBS (5 min each time with gentle rocking) and blocked with the blocking buffer consisting of 1% normal fetal bovin serum in PBS for 2 h at room temperature with gentle rocking or overnight at 4 °C. the cells were then incubated with the mouse monoclonal antibody against human PDL1 (CD274) (Invitrogen) and mouse monoclonal anti human CTLA4 (CD152) (Invitrogen)at a 1:500 dilution in the blocking buffer for 2 h at room temperature. The cells were washed twice with the wash buffer (0.05% Tween 20 in PBS, 5 min each time) and incubated for 1 h at room temperature with the secondary Goat anti-Mouse IgG (H + L), Alexa Fluor Plus 647 (Invitrogen). Then cells were washed twice with the wash buffer with gentle rocking (5 min each time) and once with PBS for 5 min then stained for 30 min at room temperature with DAPI (ThermoFisherScientific). Cells were screened by Fluorescence microscope (Olympus SC 100).

### RNA extraction and real-time quantitative reverse transcription PCR

HT-29 and Caco-2cells (10^5^ cells/well) were seeded in 6 well plates and incubated overnight. Then, miR-497@NPs and pNull @NPs, each containing an 4 μg plasmid DNA in ratio 1:1 of SPION@Ag@Cs: plasmid, was added and incubated for 48 h. The total RNA was extracted from transfected HT-29 and Caco-2 cancer cell lines, using Trizol® (life technologies) according to the manufacturer’s instructions. cDNA was obtained from 1 µg RNA (high capacity cDNA), with RevertAid First Strand cDNA Synthesis Kit (Thermo Scientific) and the incubation was performed on the gradient thermal cycler (Bio-Rad). The primer sequences of targeted genes are shown in Table 1. Each gene expression was normalized with the housekeeping gene GAPDH. qRT-PCR was carried out in QuantStudio™ 5 Real-Time PCR Instrument. TheqRT-PCR was performed in duplicate for each sample using Maxima SYBR Green qPCR Master Mix, ThermoScientific. PCR cycling condition was as follow: 95 °C for 5 min and 40 cycles of 94 °C for 15 s, annealing for 60 s, extension at 72 °C for 10 s. The 2^−ΔΔCT^ formula, the method of relative quantification of mRNA was used to determine the fold difference in gene expression^24^.

**Table 1:** Real time PCR primers.

Gene	Forward primer	Reverse primer	Accession number
P53	CCTCAGCATCTTATCCGAGTGG	TGGATGGTGGTACAGTCAGAGC	NM_001407269.1
BCL-2	TCGCCCTGTGGATGACTGA	CAGAGACAGCCAGGAGAAATCA	NM_000633.3
P21	AAGACCATGTGGACCTGT	GGTAGAAATCTGTCATGCTG	NM_001220777.2
PD-L1	TCACGGTTCCCAAGGACCTA	AGAGCTGGTCCTTCAACAGC	NM_001314029.2
CTLA-4	AGGTGACTGAAGTCTGTGCG	CATGAGCTCCACCTTGCAGA	NM_001037631.3
GAPDH	CCATGGAGAAGGCTGGGG	CAAAGTTGTCATGGATGACC	NM_001357943.2

### Cell cycle analysis

Treated cells with miR-497@NPs and pNull @NPs were washed with Dulbecco’s phosphate-buffered saline (DPBS), trypsinized using 0.05% trypsin–EDTA, fixed in 70% ethanol in DPBS, and stored at 4 °C overnight. Cells were incubated with 50 μg/ml PI (Thermo Scientific) containing 8 μg/ml RNaseAin the dark at 37 °C for 30 min. Cells were then analyzed by CytoFLEX Flow Cytometer (Beckman Coulter Life Sciences, USA) to measure the cell fluorescence (BECKMAN COULTER Inc., Cairo, Egypt and Cat. No. 4238055-CB) for cell cycle analysis. The percentage of cells in G0/G1, S, and G2/M phases of the cell cycle was calculated using CytExpert Software^25^.

### Bioinformatics studies

#### Prediction of the miR-497-5p targets

The miRBase database (http://www.miRbase.org/) was used to obtain the sequence of MiR-497-5p. After that, ThemiRWalk v2.0 database was utilized to predict a miR-497-5p target. The targets of miRNA that are produced when different prediction algorithms intersect are available on the MiRWalk server^26^.

#### Analysis of functional enrichment

Database for Annotation, Visualization and Integrated Discovery (DAVID, https://david.ncifcrf.gov/) conducted enrichment analyses for gene ontology (GO) and KEGG pathways. Corrected value P ≤ 0.05 was taken into consideration for significant enrichment^27^.

#### Protein–protein interaction (PPI) analysis of miR-497-5p target genes

Protein–protein interaction network was analyzed by STRING (http://string-db.org/)^28^, which included direct as well as indirect associations of proteins. Using Cytoscape software v 3.10.0^29^ the network figure was drawn for the selected target genes (connected with one or more genes) after evaluating the results from STRING analysis.

### Statistical analysis

All experiments were performed at least three times, and all samples were analyzed in triplicate. Results are presented as mean ± S.M.E. The statistical difference between experimental groups was assessed by unpaired Student’s t-tests using Graphpad8.0 software.

## Results

According to fundamental studies, the absorption, scattering, and emission characteristics of metal particles are closely related to their nanoscale. Therefore, while producing nanoparticles, many nanotechnologies need precise control over size and shape. The UV–visible spectra of the sample solution were obtained right after preparation in order to confirm the formation of the magnetic–plasmonic nanocomposites.

**Fig. 1:**
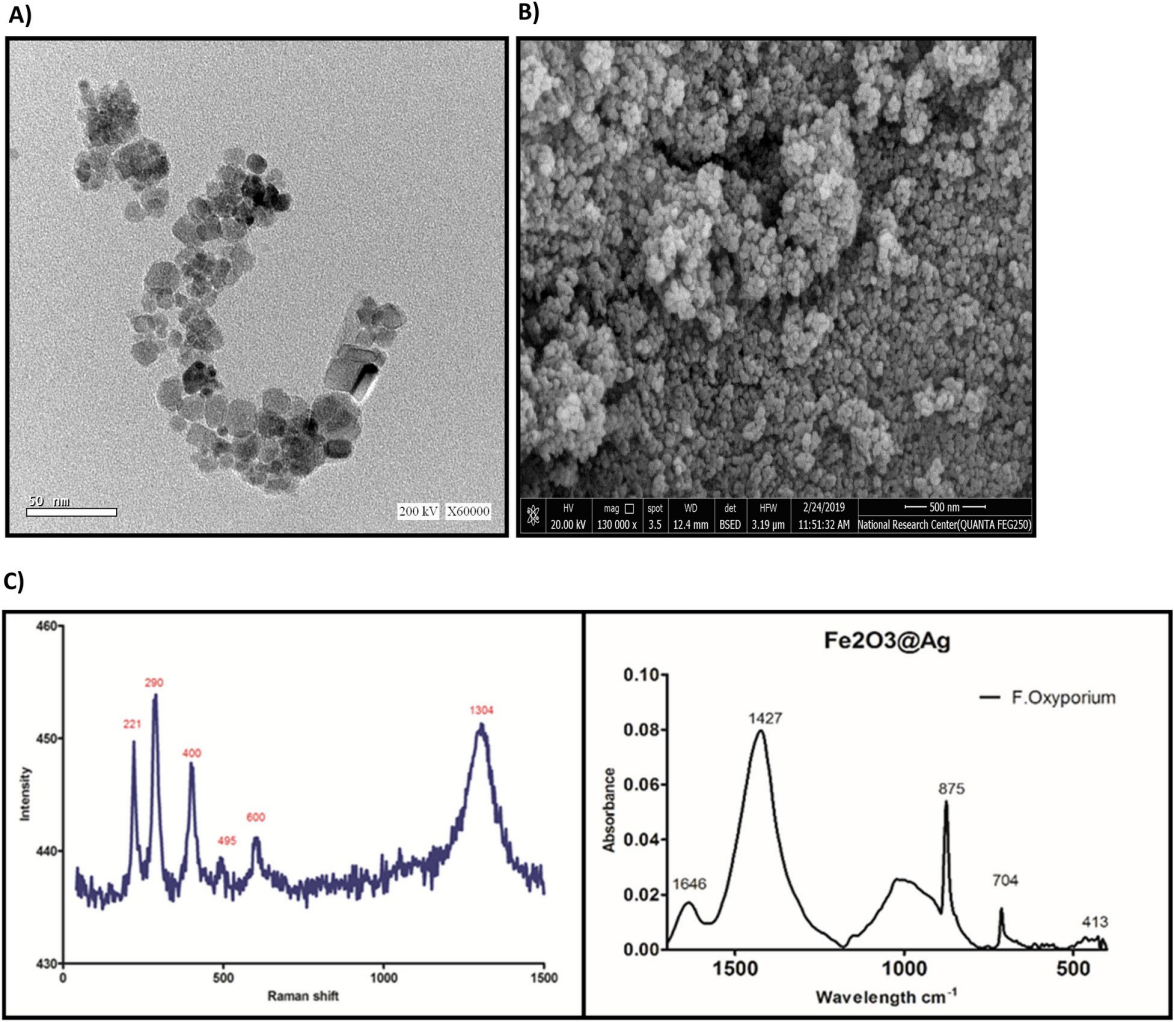
(**A**) HRTEM for the SPION@Ag@Chitosan nanocomposite, (**B**) the HRSEM of the same nanocomposite, (**C**) Raman and FTIR spectrum of the nanocomposite.

Figure 1A,B showed the HRTEM and the HRSEM respectively, the figures confirm the formation of spherical nanocomposite with particle size less than 20 nm. Figure 1C showed the Raman and FTIR spectrum of the nanoparticles formula to confirm the phase of the iron oxide and the structure of the formula. Hematite belongs to the D6 crystal space group, and the Raman spectrum should contain 3d seven phonon lines, Two A1g modes (225 cm^−1^ and 498 cm^−1^) are included, as well as four Eg modes (247, 293, 299, 412 and 613 cm^−1^). The Fe–O functional group vibrations cause separate peaks at 538 cm^−1^ and 472 cm^−1^ in the FTIR spectra of γ-Fe_2_O_3_ nanoparticles, five peaks in the range of 200–400 cm^−1^ are caused by the Eu and A2u infrared-active modes. Data showed the formation of SPION@Ag@Cs nanocomposite and that the FeO is in the form of γFe2O3.

**Fig. 2:**
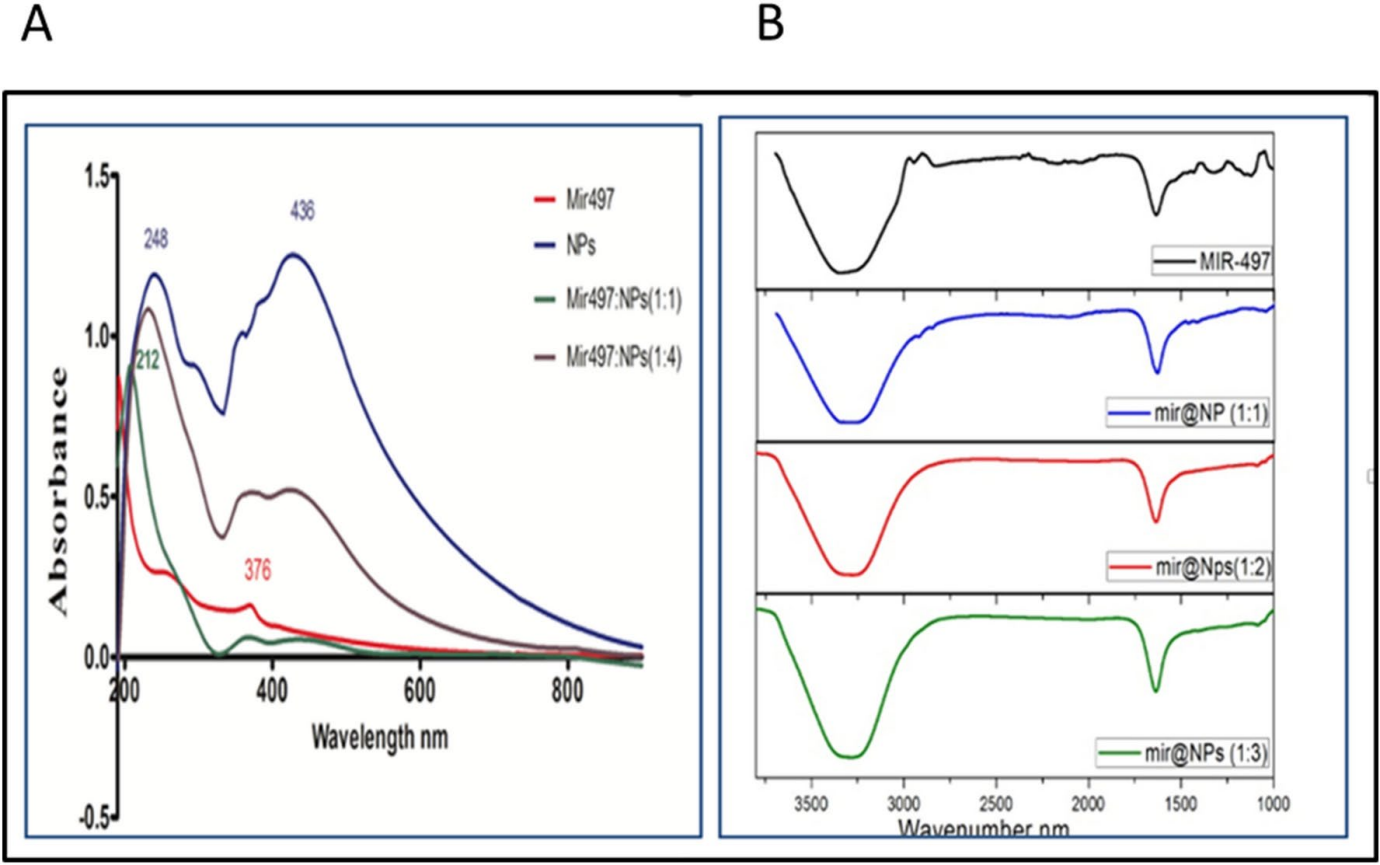
(**A**) Allocation of Mir497-5p on the SPION@Ag@CsNpsa)UV/visible. (**B**) The ATR-FTIR of the mir-497 and the mir@NPs with different ratios.

To select the suitable ratio for the nano-drug, Fig. 2a show the UV/visible spectrum of the mir-497, the SPION@Ag@Cs and the mir-497@NPs at ratios (1:1) and (1:4). Figure 2b shows the molecular structure of the mir-497, mir@NPs (1:1, 1:2 and 1:3). The data shows that the ratio n/m (1:1) in both UV/visible and FTIR spectrum demonstrate efficient loading.

**Fig. 3:**
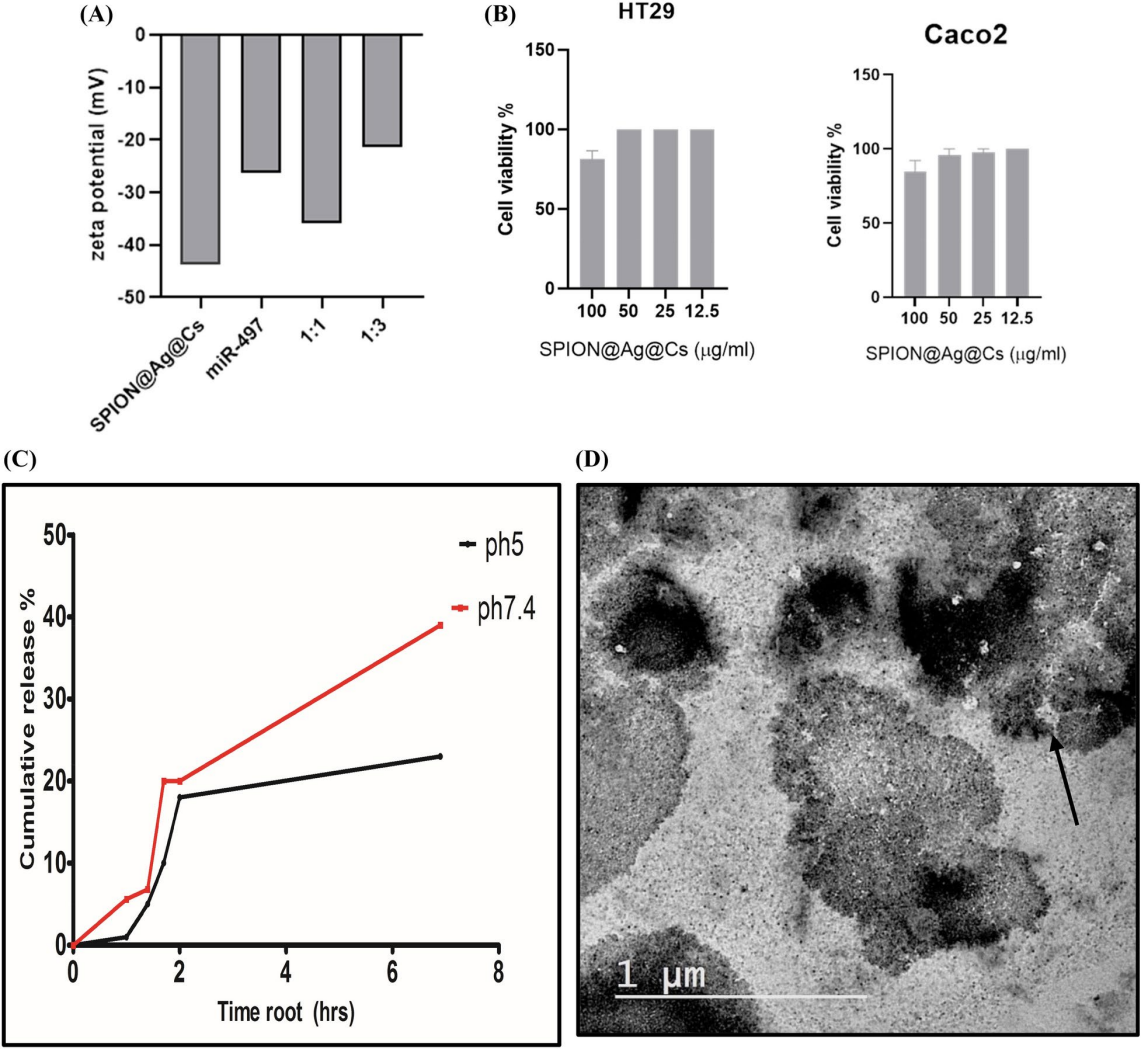
(**A**) Zeta potential of miR-497-5p, SPION@Ag@NPs and miR-497@NPS with two different ratios (1:1, and 1:3), (**B**) Cytotoxicity of HT29 and Caco2 cell lines treated with SPION@Ag@Cs at different concentrations (100–12.5 µg/ml) as measured by MTT Values are expressed as the means ± S.E.M (n = 3), (**C**) miR-497 release in PBS for pH 5 and 7.4 for 48 h, (**D**) the internalization of nancomposite in HT29 cell line the white arrow show the andosomes and the nanocomposite inside it.

Figure 3A shows the zeta potential of the mir-497, SPION@Ag@Cs, and the mir@NPs with ratios (1:1, and 1:3). The results of zeta potential confirm the selection of 1:1 ratio as it showed the best negative value for the ratio: -36 mV.

Figure 3B shows the cytotoxicity of SPION@Ag@Cs treated against HT29 and Caco2 cell lines for 48 h at different doses (100–12.5 μg/mL). The MTT assay revealed that cells treated with SPION@Ag@Cs retained their properties. Cell viability (> 80%) up to a tested concentration of 100 μg/mL indicates the high biocompatibility of SPION@Ag@Cs with living cells. Figure 3C show the drug (miR-497) release in the PBS with pH5 and 7.4. the data has been plotted for the cumulative release % (D_t_/D_total_), where D_t_ is drug concentration at time t and D_total_ is the total amount of drug loaded to the nanoparticles. The data showed that the drug release rate is higher for the ph7.4 (5) than the pH5 (3), the calculated rate kinetics was measured using the semi-empirical power law equation: D_t_/D_total_ = Kt. Figure 3D shows the HRTEM of HT29 cells after incubation with SPION@Ag@Cs NPs for 6 h, showing the nanocomposite distribution inside cells, the white arrow shows the endosome.

**Fig. 4:**
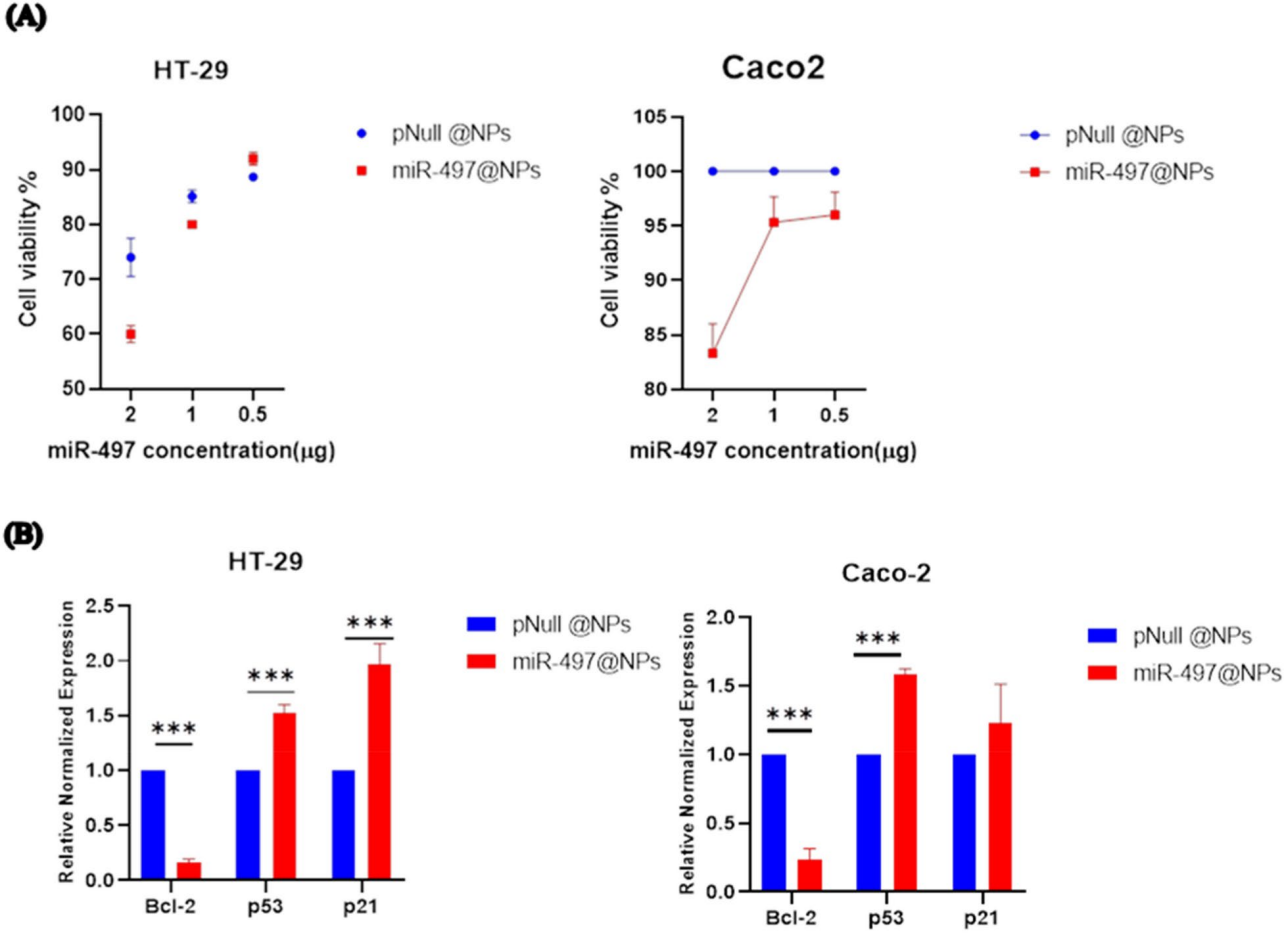
The In vitro functional validation pmiR497-@Nano composite. (**A**) Cytotoxicity of CRC cell lines transfected with pNull@Nano and pmiR497-@Nano, as measured by a MTT assay. (**B**) Relative mRNA levels of Bcl-2, p53,and p21 genes in treated cells with miR-497@NPS versus pNull @NPs against HT-29 and Caco-2 cancer cell lines. mRNA level of each gene is normalized to GAPDH level. Error bars represent S.EM, ***p < 0.001.

To examine the potential of pmiR497-@Nano composite to suppress CRC cells proliferation, HT29 and Caco2 cells were transfected with pmiR497-@Nano composite or pNull@Nano as a control. MTT assay results revealed that cell proliferation was significantly reduced upon increasing the concentration of pmiR497-@Nano composite compared to the pNull@Nano (p < 0.05) (Fig. 4A). Furthermore, we found that cancer cells treated with pmiR-497@Nano might be induce the expression levels of apoptotic genes through significant downregulation of Bcl-2 level by fold change of 0.16 and 0.2 in HT29 and Caco2 cell lines, respectively. Also, p53 was significantly upregulated in HT29 and Caco2-treated cancer cells by fold changes of 1.5 and 1.58, respectively. P21 gene expression showed a nonsignificant upregulation against the treated Caco2 cell line. However, it significantly upregulated in the HT29 cell line in response to pmiR-497@Nano treatment compared to treated cells with pNull@Nano (Fig. 4B).

**Fig. 5:**
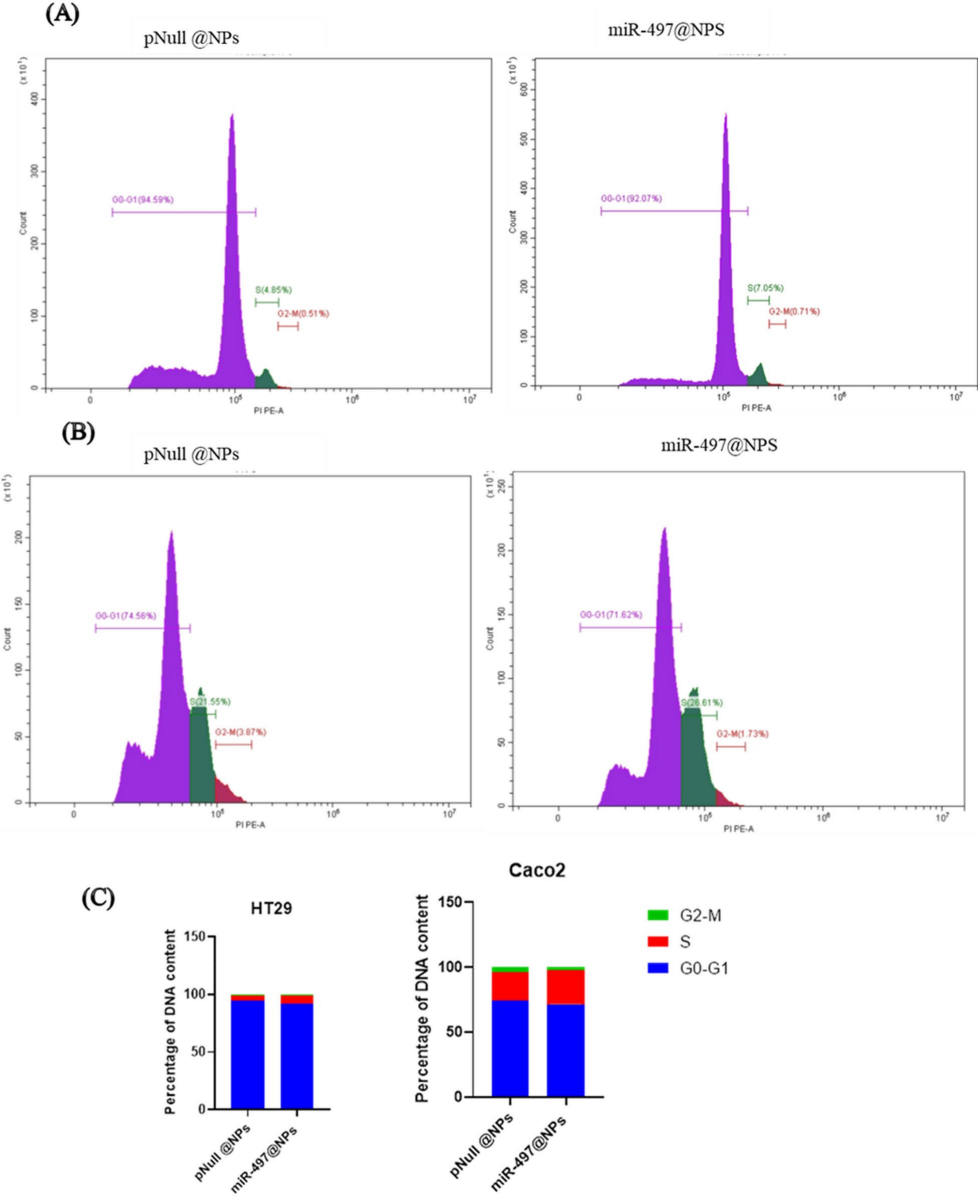
Cell cycle distribution analysis by flow cytometry in human CRC cell lines. (**A**) Representative data of flow cytometric analysis of transfected pmiR-497@NPS in HT29 cell. (**B**) Representative data of flow cytometric analysis of transfected pmiR-497@NPS in Caco2 cell. (**C**) A column chart displays the percentage of cells in each cell cycle phase in response to pmiR-497@NPS. The analysis was carried out by CytExper flow cytometer.

To explore the effect of pmiR-497@Nano on cell cycle regulation flow cytometric analyses was carried out. It revealed that the percentages of miR-497 transfected HT29 and Caco2 cell lines in S-phase were 7.05% (HT29) (Fig. 5A) and 26.6% (Caco2) (Fig. 5B) greater than those in the treated cells with pNull@Nano. These data indicated that miR‑497 reduced cell proliferation presumably by the induction of cell cycle arrest in S-phase.

**Fig. 6:**
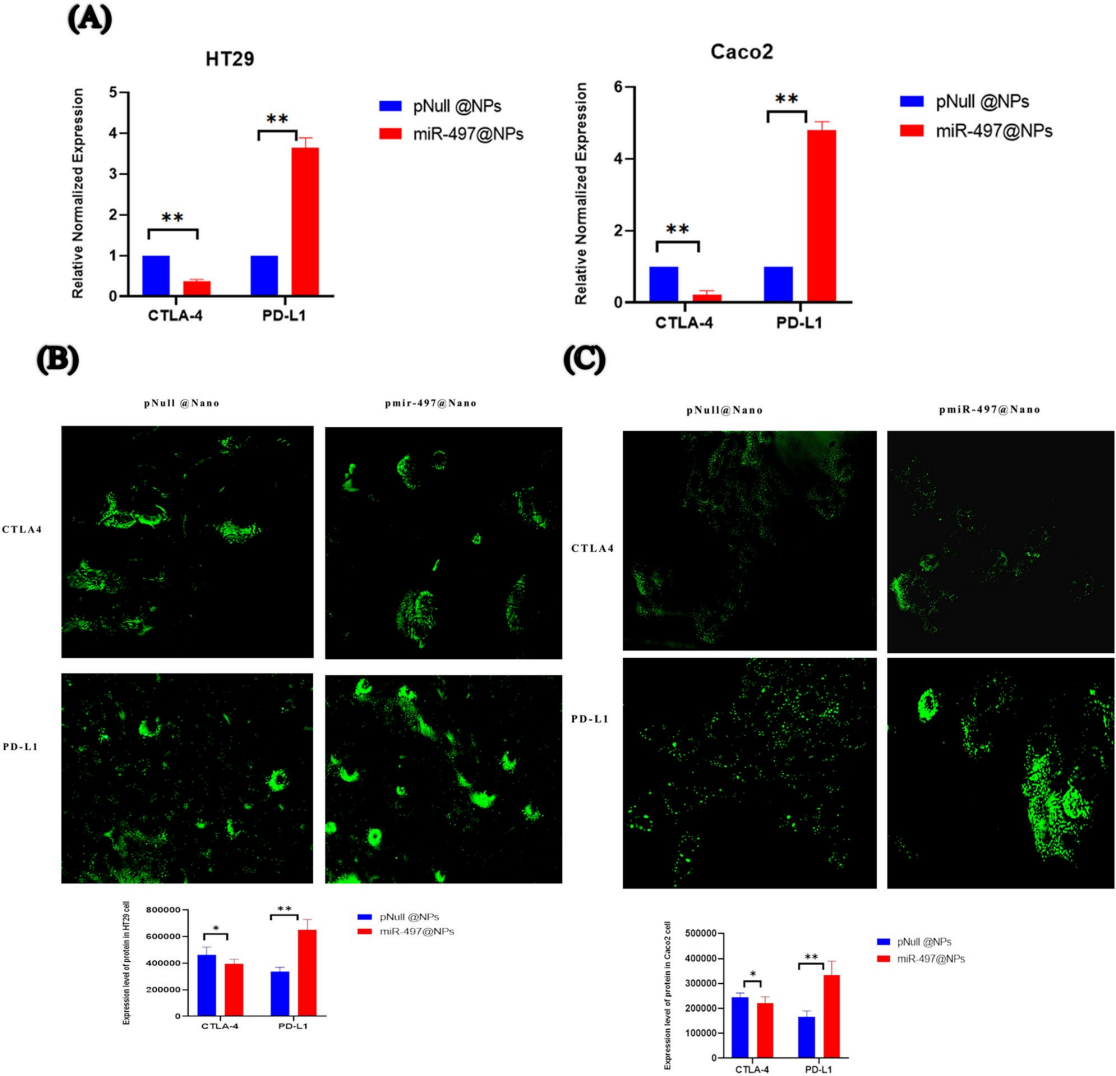
(**A**) Expression level of immune checkpoints PD-L1 and CTLA-4 in CRC cell lines. (**B**) Immunostaining analysis of immune checkpoints PD-L1 and CTLA-4 in HT29 transfected with pmiR497-@Nano. (**C**) Immunostaining analysis of immune checkpoints PD-L1 and CTLA-4 in Caco2 transfected with pmiR497-@Nano. Cells were analyzed under fluorescence microscope (× 200) and The pictures were captured with Olymus camera fixed in inverted Olympus microscope with red and green florescence filters in the range of 480–630 nm. The analyzed using image j software. Values are presented as the mean ± S.E.M,*P < 0.05, **P < 0.01.

As shown in Fig. 6A, compared to pNull@Nano, relative mRNA expression of CTLA4 was significantly downregulated in HT29 and Caco2 cells transfected withpmiR-497@Nano by fold changes (0.3769), and (0.2268), respectively (p < 0.01). On other hand, the gene expression of PD-L1 was significantly upregulated in HT29 and Caco2 cells transfected with pmiR-497@Nano by fold changes (3.64),and (4.79), respectively (p < 0.01). Consistent with the results of RT-qPCR. The immunofluorescence results showed significant downregulation of CTLA4in HT29 (Fig. 6B) and Caco2 cell lines (Fig. 6C) in response to miR-497-5p@Nano treatment. Whereas, the expression level of the PD-L1 was significantly up-regulated in response to miR-497-5p@Nano treatment against HT29 (Fig. 6B), and Caco2 (Fig. 6C) cell line.

**Fig. 7:**
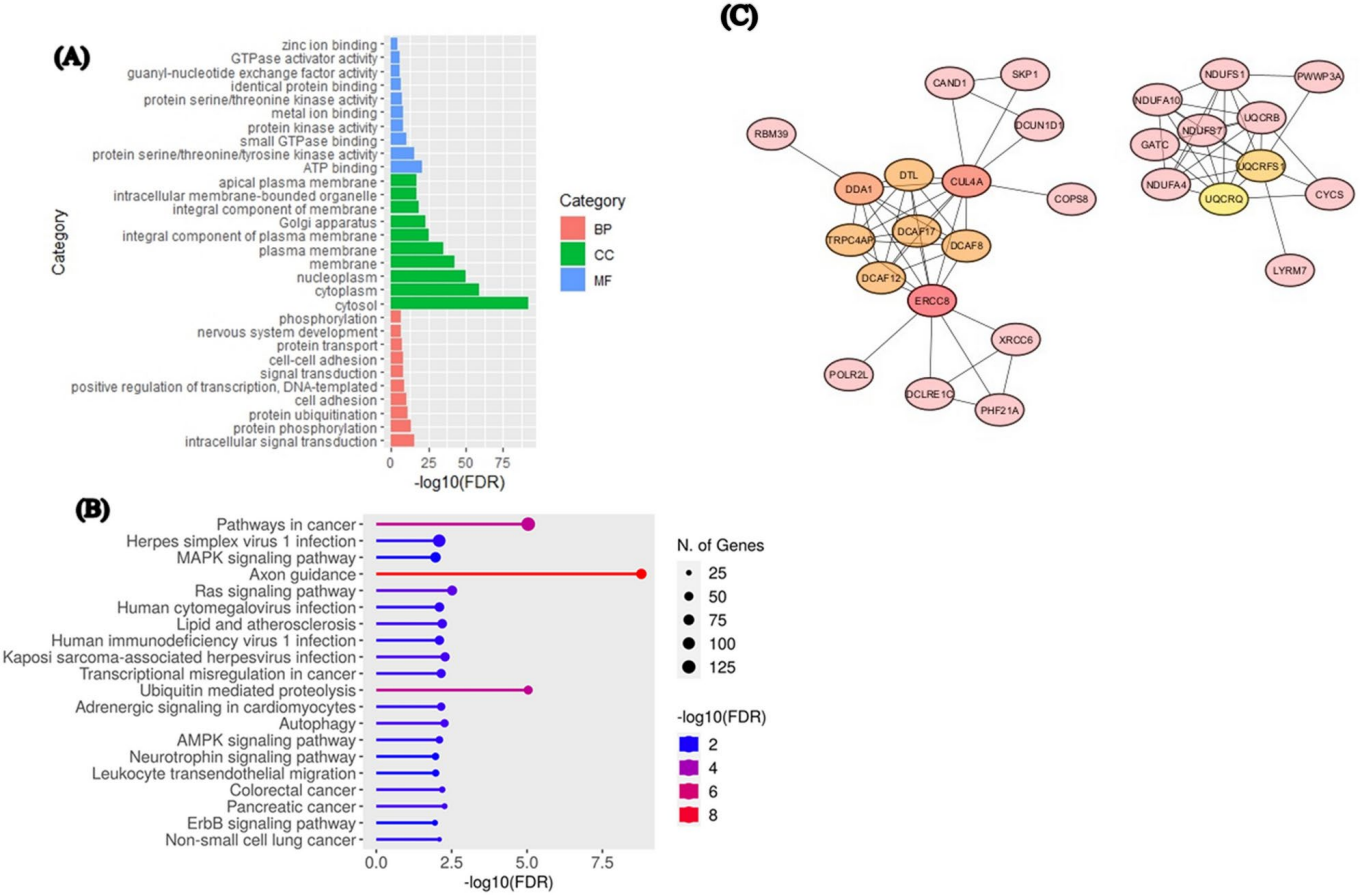
Functional study of mir-497-5p target gene enrichment. (**A**) The gene ontology are created based on the P-values related to the biological process (BP), cellular component (CC), and molecular function (MF) terms in. (**B**) KEGG pathways enrichment (p < 0.05). (**C**) Protein–protein interaction network among miR-497-5p Targets.

Gene ontology analysis of miR-497-5p Target genes was performed to understanding molecular function, cellular component, and biological process of these target genes. The most (corrected p-value < 0.05) GO terms such as intracellular signal transduction (GO:0,035,556),protein phosphorylation (GO:0,006,468), and protein ubiquitination (GO:0,016,567)were enriched in biological process. Also, cytosol (GO:0,005,829), cytoplasm (GO:0,005,737), and nucleoplasm (GO:0,005,654) were enriched in cellular component. The molecular functions of GO were enriched to protein serine/threonine/tyrosine kinase activity (GO:0,004,712), small GTPase binding (GO:0,031,267), and protein kinase activity (GO:0,004,672) (Fig. 7A). Moreover, similar to GO terms, KEGG pathway analysis revealed that Axon guidance pathway, Ubiquitin mediated proteolysis pathway, Pathways in cancer, Pancreatic cancer pathway, Non-small cell lung cancer pathway, and Colorectal cancer pathway was significantly enriched (P < 0.05) (Fig. 7B).

We found different hub nodes that includedERCC8, CUL4A, DDA1, DCAF17, DCAF8, DTL, DCAF12, TRPC4AP, UQCRFS1, and UQCRB (Fig. 7C). These candidate genes have important biological regulatory functions.

## Discussion

Globally, colorectal cancer (CRC) continues to be the third most frequent kind of cancer. The main factors that contribute to CRC have been determined to be microsatellite instability (MSI) and, inflammation^30,31^. Systemic inflammation is characterized by an increase in the production of pro-inflammatory cytokines and acute phase proteins that enter the bloodstream^32^. Our goal in this work was to develop a formula that targets the PD1/ PDL1, and CTLA-4 genes in colorectal cancer in vitro using super-paramagnetic @Silver @Chitosan nanoparticles (SPION@Ag@CS) loaded with mir-497-5p. We also investigated the impact of this treatment on the therapeutic and diagnostic aspects of CRC. We have demonstrated in earlier research that the employed nano-formula generates fluorescence, opening up new avenues for diagnosis. After studying the uptake of SPION@Ag@Cs by the colorectal cell lines HT29, and Caco2, we found that the cytotoxicity increased with the uptake, which began extremely early and continued for 72 h. On the SPION@Ag@Cs, the mir497-5p has been loaded at concentrations of 10 µg in three distinct ratios (1:1, 1:2, and 1:3). The 1:1 and 1:2 ratios enhanced the distribution of mir-497-5p onto the nanoparticles, as demonstrated by the UV/visible bands and the FTIR molecular structure of the compound both before and after loading. At a 1:1 concentration, the mir497-5p’s band at 268 nm has vanished, but the bands at 378 and 428 nm are still present. The other peaks, with a reduction in intensity, were visible at 1470, 1400, and 1050 cm-1 in the molecular structure of a 1:1 ratio. The miR-497-5p and the SPION@Ag@Cs are negative, according to the dynamic light scattering (DLS) data, which suggests that the varied ratios simply change the zeta potential value rather than the charge. It was hypothesized that miR‑497 may serve as tumor suppressors in CRC. The MTT assay demonstrated that CRC cells transfected with pmiR-497@nano exhibited reduced proliferative abilities compared with those of the control cells. Furthermore, in our in silico* study* revealed that, miR‑497 could be induced cell cycle arrest by direct targeting of some important genes associated with the S phase like Cyclin-Dependent Kinase 20 (CDK20), Minichromosome Maintenance Complex 5 (MCM5), which is essential for the formation of the replication fork and the unwinding of DNA.

In this study illustrated that pmiR497-@Nano composite dysregulated the marker of apoptotic genes Bcl2,p53, and p21. miR-497- loaded nanoparticles promotes apoptosis primarily through the intrinsic pathway by activating the Bcl-2/Bax-caspase9-caspase3 pathway^33^. This pathway involves the release of cytochrome c from mitochondria, leading to the activation of caspase-9 and subsequently caspase-3, which executes apoptosis. Also, miR-497 targeted the FAS receptor (CD95), a cell surface receptor that plays a crucial role in the extrinsic pathway of apoptosis. When FAS ligand (FASL) binds to the FAS receptor, it triggers the formation of the death-inducing signaling complex (DISC), leading to the activation of caspase-8 and downstream effector caspases like caspase-3.

Additionally, Studies have shown that p53 can induce apoptosis by upregulating pro-apoptotic genes such as BAX and downregulating anti-apoptotic genes like BCL2^34^. Also, p21, a downstream target of p53, can enhance this apoptotic response by inhibiting cell cycle progression and promoting cell death^35^. These mechanisms provide a plausible explanation for the observed effects of our pmiR497-@Nano composite.

Our bioinformatics results showed that miR-497 targeted the some key genes involved in apoptosis. Therefore, it was supposed that miR‑497 function as tumor suppressors in HCC.

Using qRT-PCR and immunofluorescence labeling, the absorption of miR-loaded NPs has been verified in the CRC cell lines Caco2, and HT29.

The glycoprotein CTLA-4, sometimes referred to as CD152, is a member of the human Ig gene group and is present on the surface of activated T cells in both humans and mice^36–38^. Numerous tumor cell lines, including those from the breast, colon, lung, ovarian, uterine, bladder, osteo/rabdomyosarcoma, neuroblastoma, and melanoma, express CTLA-4. 88% of cell lines have CTLA-4 expression at different densities, according to flow cytometry studies, with osteosarcoma and breast cancer cell lines exhibiting the strongest staining^39^. Tumor growth and metastasis can be accelerated by inhibitory immunological checkpoints, which impede anti-tumor immune responses. Decreased anti-tumor immune responses in the tumor microenvironment have been associated with the CTLA-4/CD80,CD86 axis between immune cells and tumoral cells (Derakhshani, Hashemzadeh, Asadzadeh, and Shadbad 2021).

This study has demonstrated that the expression of CTLA4 significantly decreased in response to mir497@nano therapy in HT29 and Caco2 cell lines. This finding may be explained by a bioinformatics investigation that demonstrated miR-497 specifically targeted transcription factors that controlled the expression of CTLA4, including Forkhead boxP3 (Foxp3) and the nuclear factor of activated T cells 5 (NFAT5) (Additional file 1). The transcription of CTLA-4 is stimulated when NFAT5, one of the NFAT proteins, binds to the CTLA-4 promoter. Furthermore, the altered NFAT site eliminated the activity of the CTLA-4 promoter, and NFAT inhibitors reduced the production of CTLA-4^40^. Foxp3 is an additional transcription factor that enhances CTLA-4 transcription by interacting with NFAT at the CTLA-4 promoter^41^.

It has been shown that the downregulation of the active T-cell-mediated immune response provided by PD-L1 produced by tumor cells provides an immune evasion strategy. Markers of the serrated route of colorectal carcinogenesis, including microsatellite instability, poor differentiation with medullary architecture, BRAF mutation, and a high frequency of lymphocytes infiltrating the tumor, are associated with PD-L1 expression in sporadic colorectal carcinomas^42^. Immune cell PD-L1 expression was significantly higher near the invasive front of mismatch-repair-deficient tumors, but this expression was unaffected by the different genetic subtypes of the tumors^43^. By triggering immunological checkpoints, malignant tumors can create an immunosuppressive environment. Among the well-studied immune checkpoints are programmed cell death 1 (PD-1) and PD-ligand 1 (i.e., the natural ligand of PD-1) dependent pathways. These pathways play a crucial role in both the evasion form of antitumor immunity and physiological conditions^44^. T cell-dependent mortality is increased and T lymphocyte-synthesis of interleukin-2 is suppressed as a result of the specific interaction between PD1 and PDL1. On the other hand, a recent study has shown that in renal cell cancer, miRNA-497 decrease increases PD-L1 expression. MiRNA-497 targets PD-L1, reduced cell division, clone formation, and migration^45^. Our result reveals that PDL1 showed over-expression in Caco2, and HT29 in response to Mir497@nano treatment.

These last finding could be explained by a study conducted by Yan-Jie Xu et al.^46^. They predicted that miR-497 was to be the potential target of hsa_circ_0136666. The expression of hsa_circ_0136666 and miR-497 was inversely correlated. Thus, the validation of hsa_circ_0136666 directly targets miR-497, thereby promoting PD-L1 expression. Furthermore, they concluded that hsa_circ_0136666 regulate tumor immunity by sponging miR-497 and Treg activity in colorectal cancer. Additionally, they disclosed that one possible treatment approach for colorectal cancer is to target the hsa_circ_0136666/miR-497/PD-L1 axis.

Understanding the biological basis of intricate characteristics requires an analysis of the PPI network. In our study, we identified the top 3 hub genes that targeting for miR-497including ERCC8, CUL4A, and DDA1.

According to the MCC method, Excision repair 8 (ERCC 8) was shown to be one of the hub genes with the highest ranking. ERCC 8 is one of the excision repair cross-complementation (ERCC) genes which involved in DNA repair. Further evidence supporting the involvement of ERCC 8in the carcinogenesis of colorectal cancer was presented by Matejcic et al.^47^. They studied the genetic variant in this gene associated with the risk of colorectal cancer.

Cullin 4A (CUL4A) protein participates in the turnover of important regulatory proteins by assembling different E3 ubiquitin ligase complexes^48^. During chaperone-mediated ubiquitylation, CUL4A is crucial for physiological functions including cell survival, development, growth, and cycle^49^. In the meantime, all throughout the carcinogenesis process, it may interact with associated genes. Various types of cancer that overexpress CUL4A have been identified, such as hepatocellular carcinoma^50^, primary malignant pleural mesothelioma, primary human breast cancer, prostate cancer, and epithelial ovarian tumor^51,52^. A study conduct by Yi et al.^53^ reported that CUL4A is highly expressed in Colon Cancer (CC) and promotes the proliferation and inhibits the apoptosis of CC cells by regulating the Hippo pathway.

DDA1, an evolutionarily conserved gene located at 19p13.11, could have a role in nuclear factor kappaB (NFκB) activation. Zhao et al.^54^ investigated whether DDA1 activates the NFκB pathway, which may lead to carcinogenesis and the advancement of stage II colon cancer. They found that in patients with stage IIB–IIC colon cancer, positive expression of DDA1 or co-expression with p65 nuclear translocation was associated with a higher risk of tumor recurrence. In colon cancer lines, overexpression of DDA1 promoted cell cycle progression and enhanced cell proliferation. In this study, we reported that 3 hub genes and transcriptional regulator factors could be regulated functional and specific gene in colorectal cancer by targeting miR-497 /CTLA-4 axis.

## Conclusions

In the in vitro investigation, the 1:1 and 1:2 miR-497-5p:SPION@Ag@Cs ratio has shown great promise. Following a 48-h incubation period, our findings verified the transfection of colorectal cell lines under investigation by miR-497-5p using the nanocomposite SPION@Ag@Cs. The downregulation of CTLA4 in response to the therapy and the overexpression of PDL1 in both HT29 and caco2 are also confirmed by the results. The two cell lines, HT-29 and Caco-2, are both produced from intestinal cancer. HT-29 differentiates into mucus-secreting cells, while Caco-2 becomes an absorptive kind of cell, the two main cell types in the intestine. As a result, the markers of distinction are likewise unique. Our results also revealed that targeting the miR-497/CTLA4axis is a potential immunotherapy treatment strategy for colorectal cancer.

## Supplementary Information

Supplementary Information.

## Data Availability

The datasets used and/or analyzed during the current study are available from the corresponding author on reasonable request. Real time PCR prime accession number are available in Table 1. P53. NM_001407269.1; BCL-2NM_000633.3; P21NM_001220777.2; PD-L1NM_001314029.2; CTLA-4NM_001037631.3; GAPDHNM_001357943.2.

## Abbreviations

SPION: Superparamagnetic iron oxide nanoparticles
NPs: Nanoparticles
miR: MicroRNA
CRC: Colorectal cancer
CTLA4: Cytotoxic T lymphocyte associated antigen 4
PDL1: Programmed death ligand 1
